# The Recovery Narrative: Politics and Possibilities of a Genre

**DOI:** 10.1007/s11013-019-09623-y

**Published:** 2019-03-21

**Authors:** Angela Woods, Akiko Hart, Helen Spandler

**Affiliations:** 1 Institute of Medical Humanities, Durham University, Caedmon Building, Leazes Road, Durham, DH1 1SZ UK; 2 Mind in Camden, Barnes House, 9-15 Camden Rd, London, NW1 9LQ UK; 3 School of Social Work, Care and Community, University of Central Lancashire, Preston, Lancashire, PR1 2HE UK

**Keywords:** Recovery, Mental illness, Narrative, Genre, Critical medical humanities

## Abstract

Recovery is now widely acknowledged as the dominant approach to the management of mental distress and illness in government, third-sector and some peer-support contexts across the United Kingdom and elsewhere in the Anglophone Global North. Although narrative has long been recognised in practice and in policy as a key “technology of recovery,” there has been little critical investigation of how recovery narratives are constituted and mobilised, and with what consequences. This paper offers an interdisciplinary, critical medical humanities analysis of the politics and possibilities of Recovery Narrative, drawing literary theoretical concepts of genre and philosophical approaches to the narrative self into conversation with the critiques of recovery advanced by survivor-researchers, sociologists and mad studies scholars. Our focus is not on the specific stories of individuals, but on the form, function and effects of Recovery Narrative as a highly circumscribed kind of storytelling. We identify the assumptions, lacunae and areas of tension which compel a more critical approach to the way this genre is operationalised in and beyond mental health services, and conclude by reflecting on the possibilities offered by other communicative formats, spaces and practices.

## Introduction

It’s the mid-2010s, and the Country Valley Mental Health Trust has appointed a new Chief Executive to oversee implementation of its recovery strategy. Samira is charged with the not inconsiderable task of shifting the organisation from its predominantly symptom-focussed medical model approach towards being person-centred and recovery oriented. She begins by asking colleagues already working with a recovery model what kind of training, initiatives and policies helped transform their thinking and practice. The response she receives is clear and consistent: listening to people’s narratives of recovery. Organisational change, she concludes, must begin with individuals’ stories. Partnering with the local Recovery College, Samira sets up a five-day programme in which service-users work to produce, refine and rehearse their own systematic recovery narrative. A select few then get paid positions through which to share their narratives with professionals in a range of contexts – from the induction of new staff, to training of early intervention in psychosis teams, to meetings of the Board of Governors. Of all the initiatives implemented as part of the recovery strategy, Samira says that it is the stories of these experts by experience which have had the biggest impact.

*

It’s the mid-2010s, and Ben is a young person struggling with unusual experiences. When he becomes part of a dynamic mental health charity, his world transforms. He grows in confidence and is inspired to help make a difference. He starts to speak publicly about his experiences at conferences and on the radio and is extremely successful in doing so. So successful that he becomes an ambassador for a major national anti-stigma campaign and, for a while, the voice of young voice-hearers. But a few months, maybe a year down the track, the invitations and engagements dry up. His story is already in the public domain, and the charities, journalists, TV and radio producers want fresh faces. From hospital, where he has been sectioned, he speaks of a painful ebbing away of his sense of self-worth.

*

Deliberately, we start with stories about stories. Samira and Ben are not anonymised participants in an empirical study, but they are also not unknown to us. Their stories are real, although we have changed some details. We offer these stories to focus attention on the context and mode of narrative production, and to challenge the assumed transparency, neutrality and compulsory positivity of the Recovery Narrative.

This article is concerned with how experiences of madness, distress and mental illness are represented and shared late in the second decade of the twenty-first century. More specifically, our aim is to offer a critical analysis of the Recovery Narrative as one particular modality for the transmission of such experiences. We use capital letters and the singular “Narrative” advisedly, distinguishing the Recovery Narrative as an overarching category or type from the myriad of individual testimonies which speak of recovery in the context of mental distress and illness.

We begin by addressing the wider context of recovery from our vantage-point in the United Kingdom in 2019. Although narrative has been recognised as a key “technology of recovery” (Smith-Merry, Freeman, and Sturdy 2011) there has been little critical investigation of how narratives of recovery are constituted and mobilised, and with what consequences. We analyse the Recovery Narrative as a particular kind of story produced within specific sites: commissioned by or facilitated within mental health services; championed by charities and in mental health campaigns; presented formally at mental health conferences; and promoted by alternative or activist movements.^1^ Within these contexts, the Recovery Narrative can present what at face value might appear to be very different accounts of recovery, including recovery from mental illness (best achieved through compliance with psychiatry, acceptance of biomedical models, adherence to clinical care plans) and recovery from past experiences of trauma (best achieved independently or through the rejection of psychiatry). While the latter is mobilised within contexts like the Hearing Voices Movement as an explicit challenge to the former (Romme et al. 2009), and their epistemological, ontological and political differences are the subject of extensive analysis and debate, far less attention has been paid to the fact these stories are articulated through a common framework. Our interest goes beyond the thematic contents of these stories to the structures of their intelligibility; our focus is not on the specific accounts of individuals, but on the Recovery Narrative as genre. This article analyses the form, function and effects of the Recovery Narrative as a highly circumscribed kind of storytelling, identifying the assumptions, lacunae and areas of tension which demands a more critical approach to the way it is operationalised, in and beyond mental health services. We conclude by reflecting on the possibilities offered by other communicative formats, spaces and practices.

Working within the critical medical humanities (Whitehead et al. 2016), we primarily draw on literary and sociological approaches, as well as experiential knowledge gained from our involvement in mental health activism, publishing, campaigning and policy in the UK and internationally. We have engaged extensively with individuals and communities for whom what is cast as mental illness is a significant if not defining part of life, including as facilitators, family members and allies. We do not call for a more critical engagement with Recovery Narrative ignorant of what is at stake personally, ethically and politically. Acknowledging the terrain is difficult and our navigation of it likely to be imperfect, we hope that what follows can help not only to reframe debate about what constitutes a good, effective or politically acceptable narrative (and experience) of recovery, but also to encourage the proliferation of alternatives.

## The Place of Narrative Within Recovery Policy

Whether as a movement, model, framework or guiding ethos, recovery names an approach to severe mental illness which is now “the hegemonic guiding principle of public mental health policy” (Braslow 2013:783) in the majority English-speaking countries of the Global North. Much has been written about how recovery stems from the consumer/survivor/ex-patient movements of the 1970s and 1980s (Deegan 1988), and has been “mainstreamed” or “co-opted” by mental health professionals and services since the early 2000s (Repper and Perkins 2003; Davidson et al. 2005; Amering and Schmolke 2009; Perkins and Slade 2012; Braslow 2013). It is common for champions and critics alike to highlight the lack of consensus regarding what constitutes recovery (Mental Health “Recovery” Study Working Group 2009; Pilgrim 2009; McCranie 2011) while simultaneously affirming William Anthony’s account of it as “a deeply personal, unique process of changing one’s attitudes, values, feelings, goals, skills and/or roles…a way of living a satisfying, hopeful, and contributing life even within the limitations caused by illness” (Anthony 1993:21).^2^ Being “deeply personal,” recovery has been difficult to define, measure and operationalise (Bellack and Drapalski 2012) even while being the focus of intensifying attention, analysis, action, and, increasingly, critique.

The lack of consensus over the definition of recovery, combined with an ongoing debate about whether it is or should be a “top-down” policy directive or “bottom-up” survivor-led programme of action, has focussed considerable energy and research capacity on identifying and isolating its constituent components. The production of scales, measures and typologies (Corrigan et al. 1999; Andresen, Caputi, and Oades 2006; Drapalski et al. 2012; Killaspy et al. 2012) has now progressed to systematic reviews and narrative syntheses (Leamy et al. 2011; Tew et al. 2012; Slade et al. 2012; Scheyett, DeLuca, and Morgan 2013; Drake and Whitley 2014; Temesgen, Chien, and Bressington 2018); mechanisms by which the mainstreaming of recovery is further reinforced. “Recovery is everywhere” (Rose 2014, 217) and even if conceptually it is still a “mélange of beliefs and values” (Braslow 2013:783), service users and practitioners have certainly *felt* the effects of recovery policies, practices and discourses as they have come to dominate the mental health landscape since the 1990s.

Although there is no shortage of research and evaluation of recovery, its emergence and increasing power within the mental health sector is not solely evidence-driven. As McWade (2016) has argued, recovery is not necessarily a “thing” that can be co-opted or mainstreamed; various enactments of recovery have been brought into being through social and material practices which suit different agendas at different times. Nonetheless, beneath continued contestation about what recovery is, or should be, it is possible to identify a distinct policy “story-line” which animates current recovery policies, practices and discourses (Pilgrim 2009; Rose 2014). Story-lines, suggests Maarten Hajer in his influential study of modern environmental policy, “are narratives on social reality” which combine “elements from many different domains” and “provide actors with a set of symbolic references that suggest a common understanding” (Hajer 1997:62). Drawing on Hajer’s work, Catherine Needham’s analysis of personalisation as the overarching story-line of public services emphasises its elasticity, emotional resonance and openness to interpretation, qualities which have enabled a wide range of divergent interests to sign up to and advance it without needing to reconcile internal tensions. Crucially, for our purposes, Needham highlights the central role of individual testimonies in articulating, legitimating and achieving policy aims:

A key feature of the personalization story-line is that formal policy evaluation is backed up by powerful stories of individual transformation: case studies and testimonies are regularly deployed in government documents and reports from other organizations promoting personalization. A senior member of [the social innovation network] In Control explains that stories have been a key part of promoting personalization: “One of the things that we did very early on was start to tell positive stories about self directed support and how it was working, and that’s what’s captured the imagination. That’s what sells newspapers. That’s what people are really interested in.” (Needham 2011, 57)

We suggest that the success of recovery-as-policy (McWade 2016) similarly rests on the central role assigned to individual narratives of recovery. Smith-Merry, Freeman and Sturdy’s (2011) study of the implementation of a recovery approach within the Scottish mental health system is a clear illustration of this. Drawing on policy documents, as well as data collected through interviews with representative stakeholders and professionals within the sector, they argue that change has been brought about through the dissemination of “recovery technologies,” defined as “various kinds of techniques, practices and instruments that embody and instantiate the values of recovery, and that provide a means of enacting those values within the mental health system” (Smith-Merry, Freeman, and Sturdy 2011:2). Alongside the Scottish Recovery Indicator, Wellness Recovery Action Planning and peer support, participants identified Recovery Narratives as a key and arguably primary recovery technology. This was no accidental stumbling upon the power of storytelling. Modelling their work on a New Zealand initiative, the Scottish Recovery Network undertook a narrative research project to collect, publish and disseminate the recovery stories of 64 people from across Scotland (Brown and Kandirikirira 2007). The collection of stories was intended to personalise recovery, inspire hope, offer practical strategies to individuals, and constitute evidence that “recovery works.” It functioned, too, to legitimise the local policy initiative (both by connecting it to an international peer movement and by imparting it a distinctively Scottish character)^3^ and to support professionals increasingly encouraging service-users to “‘write their own story’ as part of their journey to recovery.” Smith-Merry, Freeman and Sturdy conclude from this example that:

narrative work has become an established technology in ‘recovery-oriented’ mental health services, and works to instantiate and exemplify the concept of recovery in the mental health system in a number of ways. As individual narratives are created by service users and used as a therapeutic tool by practitioners, so the practice and values of recovery are implemented, reproduced and incorporated into the institutional knowledge of the mental health services (2011, p4).

Recovery Narratives do not appear spontaneously: as technologies of recovery within mental health services they are actively solicited, circulated and mobilised in ways intended to benefit service-users, professionals and services. On this reading, it would be difficult to underestimate the centrality of narrative to the way recovery is enacted, recorded, evidenced and legitimated. Later, we explore the formal and rhetorical features of these narratives (something conspicuously lacking from Smith-Merry, Freeman and Sturdy’s study), arguing that the efficacy of this technology depends upon tight adherence to generic conventions which are laid bare in the proliferation of “how to tell your recovery story” guides and training programmes delivered in mental health settings and Recovery Colleges (Perkins et al. 2012; Nurser 2017). Before proceeding, however, we need briefly to consider the place of the Recovery Narrative within wider contestations of recovery policy.

Recovery approaches, policies and practices are becoming the focus of increasing critique from sections of the psychiatric survivor movement (“Recovery in the Bin” 2018) as well as more mainstream proponents of recovery (Davidson et al. 2006). The survivor-led critique rests on the idea that recovery was a radical idea which has been co-opted by mainstream policy-makers in order to pursue a neoliberal agenda. Researchers and activists have highlighted the complex ways in which recovery discourse is entangled and imbricated with wider policy imperatives, such as reducing welfare spending, curtailing commitment to long term social care and promoting “back to work” agendas (Esposito and Perez 2014; Friedli and Stearn 2015; McWade 2016). Recovery is also critiqued as normalising individualism, disregarding the social relations in which we are embedded, and deflecting attention from systemic inequalities and social injustice, such as racial and socio-economic discrimination (Friedli 2010; Harper and Speed 2014; Rose 2014). This malaise around recovery is also shared by some of its proponents, who express reservations around its generalisability across different cultures and also about the variability of its implementation (Slade et al. 2014). Noting that the idea of recovery as a survivor-led movement sits uncomfortably with services delivered centrally by statutory providers, Perkins and Slade (2012) question the political will as well as practical ability to divert resources to alternative, peer-led and non-statutory services.^4^

Within the context of these critiques, discussion of recovery as *narrative* has been markedly more marginal and careful. Lynne Friedli (2010), Lucy Costa et al. (2012) and Jijian Voronka (Voronka 2016a, b, In Press) offer powerful accounts of coercion and compulsion in the production of Recovery Narrative, highlighting the potential for *dis*empowerment in “telling your story” in order to meet the demands of mental health providers, and we will return to their work in more detail below. But if, as we have argued, the Recovery Narrative plays a central and enabling role within the conceptualisation and implementation of recovery, where does this wider reticence to question it come from?

One answer might be that while it seems acceptable to judge Recovery Narratives commissioned by mental health providers against a certain set of criteria (for example, the extent to which they are sufficiently efficacious in achieving individual and institutional outcomes, or sufficiently representative in demographic terms of the users of services), critiquing Recovery Narratives stemming from survivor activist movements is more fraught. The Recovery Narrative has become a deeply divisive issue amongst survivor activists. While it has been a primary vehicle for trauma-focused, narrative-driven activism (especially within contexts like the international Hearing Voices Movement (Romme et al. 2009; Coleman 1999; Dillon 2011)) it has also been strenuously disavowed by other activists who, for example, see the imperative to narrate traumatic experiences as another form of oppression. “We believe being made to feel like you have to tell your ‘story’ to justify your experience is a form of disempowerment, under the guise of empowerment” argue the UK collective Recovery in the Bin (Recovery in the Bin n.d.). As we will show, *any* critical interrogation of the Recovery Narrative grapples with thorny issues around permission and power (Cresswell and Spandler 2013; Russo 2016; Fitzpatrick 2016b). What we want to avoid are false bifurcations between professional and survivor-led Recovery Narratives, which might be different in terms of their foci and thematic contents but share key attributes in terms of form and delivery; each justifying, enacting and serving their own recovery ideals.

The Recovery Narrative is emotionally charged: indeed, that is its power and its purpose. It emerges from a place of intense suffering, and it requires emotional labour to produce and perform. It is also tied into individuals’ lives, their hopes and their pain, and is enveloped in discourses around empowerment. Our aim is not to invalidate, silence or call into question individuals’ accounts of their passage through extreme distress. Nor is this about holding the Recovery Narrative up to particular standards—whether aesthetic, academic, clinical or political. We believe that it is possible to critique the Recovery Narrative as a genre without resorting to personal critiques of individual meaning-making.^5^ Moreover, we argue that it is precisely the framing of Recovery Narrative as a genre which allows us to recognise some aspects of the labour of that meaning-making, to see and to challenge dominant forms of self-presentation within mainstream mental health and many survivor contexts. Denaturalising the Recovery Narrative we hope will call attention to the existence of many other kinds of stories and modes of self-representation, and so encourage engagement with a greater variety of formats for articulating a plurality of experiences.

## Recovery Narrative: Form, Function, Effect

While the stories told by individuals about their experiences of madness, mental illness and distress are immensely diverse, only a narrow subset of such stories are socially, professionally and politically recognised as being Recovery Narratives. Or, to put it differently, the stories which are publicly heralded as Recovery Narratives are not marked by their diversity, thematic idiosyncrasy or formal experimentation. What, then, counts as a Recovery Narrative? Before proceeding, we must clarify what we mean by the term genre, not least to dispel any suspicion that by identifying a text or a talk as making use of a genre we are in some sense declaring it to be inauthentic, fictional, or formulaic. According to literary and cultural theorist John Frow, “genre matters” because “its structuring effects are productive of meaning” and “central to the organisation of knowledge” (Frow 2005:10,4). Indeed, so central is genre “to human meaning-making and to the social struggle over meanings” that Frow argues that “*no* speaking or writing or any other symbolically organised action takes place *other* than through the shapings of generic codes” (Frow 2005:10).

Some of these generic codes are laid bare in the “Sharing your Story” guides produced by major mental health charities in the UK, USA and Canada (Mind UK n.d.; Boll 2015; Mental Health Commission of Canada 2017; Substance Abuse and Mental Health Service Administration 2017). Offering straightforward step-by-step advice for the production and dissemination of Recovery Narratives in writing, in person or through digital media, these publications give a consistent account of what individuals should be aiming for: stories which in formal terms are short (2–5 min, or 250–375 words), have an obvious beginning, middle and end, and use clear and accessible language (Substance Abuse and Mental Health Service Administration 2017). As well as being carefully crafted, often through the framework of a “journey”, they must be true and true to the individual (“Stories are powerful if they are honest and real…A thoughtful and organized story allows for a smooth delivery. It will also give your story a polished and truthful feel” p24); told “from a strong foundation of recovery”; and contain “messages of hope or a ‘call to action’” (pp. 24–25). These how-to guides for individuals bear striking similarity to accounts of the co-crafting of Recovery Narrative within clinically oriented contexts (Murphy 2007; Rudnick et al. 2011) as well as within the survivor movement as exemplified in the introduction to *Living with Voices: 50 Stories of Recovery* (Romme et al. 2009).^6^

Because the Recovery Narrative is intended for public consumption, it is often performed and, as with the editing of written narratives, the platforms through which the narrator and audience are brought together are carefully constructed. A prime example of this is the individual testimony presented at the start of many mental health conferences. The slot the storyteller is given, the time-frame she is allocated, the context in which her experience is framed, the support she has received, the position she holds, the willingness for both commissioner and audience to listen are already constituent features of the Recovery Narrative before a word has been spoken. The extent to which genre-determined expectations are fulfilled, disappointed or deliberately subverted may depend on a number of factors: Is this the first time she has told her story publicly? Has she been formally trained (for example through a Recovery College programme), or coached or mentored to focus on some parts or themes over others? Is she already well-known to the organisers or the audience? Does her story suit the prevailing idea of what recovery should be within a particular setting?

In previous work, we have used Frow’s analysis of genre as a structure of intelligibility to argue that the “First Person Accounts” of psychosis published in *Schizophrenia Bulletin* constitute a “genre of insight” (Woods 2012b). According to Frow, one of the key organising dimensions of genre is “the ‘structured situation of address’ between author and reader, a structure that refers to the power relations between speakers as well as the effects of ‘credibility, authority, and emotional tone’ created by these relations” (Woods 2012b:43). Whatever their chosen topic or theme, the First Person Accounts published in *Schizophrenia Bulletin* function to establish a specific kind of authorial credibility, that of “insight.” In clinical settings, a patient is said to have “insight” if they recognise themselves as mentally ill and requiring treatment (Amador 2004); in leading clinical and scientific journals, so Woods suggests, the inclusion of first-person narratives is conditional upon “the exclusion of *anything*, even the fictional or fanciful, which might be perceived as in any way symptomatic of schizophrenia” (2012b:44). The textual performance of insight in this context demonstrates that the author possesses a particular form of knowledge about their experience, one which acknowledges and affirms their status as a subject deserving of clinical attention and intervention, and, moreover, is *not* in possession of other forms of knowledge or making use of other modes of expression which might call that into question. The implied contract between the narrator and reader structurally reproduces that of patient and clinician: asserting continuity (a shared discourse, a common clinically meaningful vocabulary) while simultaneously reinforcing at multiple levels the hierarchies typically embedded within that relation.

Structurally, the Recovery Narrative belongs, we will argue, to this “genre of insight.” By this we do not mean that all instantiations of this genre testify to the individual’s uncritical acceptance of and compliance with psychiatric diagnosis and treatment (though of course some do). Rather, we suggest that a defining feature of the genre is the establishment of a particular relationship between narrator and reader; one in which the narrator is positioned as seeking recognition from the Other that the knowledge they possess about their own experiences qualifies as “insightful.” In this sense, even where they explicitly reject clinical authority and position psychiatric diagnoses and practices as something *from which* to recover, the Recovery Narrative within the survivor movement enacts the same appeal for recognition: it seeks confirmation from its audience that the knowledge its narrator possesses about her experience (for example, that it is a meaningful response to significant personal trauma) is true. Conversely, narratives which are not seen as “insightful,” which come across as chaotic in form or delivery, or (still) express seemingly irrational or intelligible beliefs, for example, in aliens or telepathy, struggle to be heard. This is clearly problematic from a Mad Studies perspective which seeks to problematise rationality as the main arbiter of knowledge (Russo and Sweeney 2016). The capacity for the Recovery Narrative to provide a basis upon which group identities can be articulated and consolidated rests, not only on the ability of the narrative to demonstrate insight and intelligibility, but also on the underlying context in which the story is received. Ultimately, this is a relational pact: it is the audience who recognises the story as a Recovery Narrative.^7^

The Recovery Narrative can document many different ways of understanding and framing the nature and origin of mental distress (including in relation to trauma, biological illness and/or personal crisis), suggest multiple pathways to recovery (including therapy, medication, familial or peer support, religious counselling, and mental health activism), and give varying accounts of the depth of transformation (ranging from being “symptom-free” and “back to normal”, to “living well with illness and disability”, to accepting and celebrating experiences framed as unusual). As a genre, it confers power to the reader/audience by soliciting a two-fold confirmation: first, that the narrator does indeed possess insight into her own mental distress, and second, that this insight has been hard-won through the shedding of false (erroneous, delusional, ideological or otherwise unhelpful) beliefs. The Recovery Narrative therefore functions as evidence, testifying to an individual’s experience of recovery as something which has already been achieved, at least in part. But at the same time it functions as enactment, a way of materialising recovery in the shared moment of the present. As the “Resources to Recover” web site puts it: “Sharing your story makes recovery real. It’s not a story of recovery until you tell someone else. Until that happens, it is just a hope inside you” (Boll 2015). If the Recovery Narrative conforms to a “genre of insight,” deferring to the listener/reader as the final arbiter of its truth, it must also be considered a “genre of inspiration,” securing its value and status by being emotionally uplifting, palpably reassuring, and inspiring change of some kind. The Recovery Narrative is goal-oriented and driven by a strong moral imperative, as the US Substance Abuse and Mental Health Services Administration (SAMHSA) guide to digital storytelling again makes clear:

Why should you share your story? Because:

- It helps to reduce negative attitudes and stereotypes,
- It may encourage others to seek help, and
- It can be a healing and empowering experience for you, too. (2017:3)

The Recovery Narrative is mobilised to further a range of different goals—in anti-stigma and fundraising campaigns; in clinical education, in the reform of mental health policy and practice; in promoting particular therapeutic approaches; in realising the political aims of survivors and activists—precisely because it is regarded as efficacious in inspiring change of some kind. In this, self-expression in the specific contexts in which this genre operates is highly circumscribed, goal-directed and carefully crafted to fulfil larger imperatives.

What aspects of the experience of madness, mental illness and extreme distress are elided or occluded from the Recovery Narrative? What happens to the testimonies and stories which fail to conform to the genre of insight and inspiration; those experiences which are not, for various reasons, narrativised in this way and are therefore not recognised as Recovery Narrative? In what follows, we further interrogate the Recovery Narrative by exploring four of its underpinning assumptions: that it is desirable for people to articulate their experience of madness and distress in particular narrative forms; that sharing a Recovery Narrative is largely beneficial for the storyteller; that it necessarily has wider societal benefits; and, finally, that Recovery Narrative should not be the focus of critique. These unspoken tenets of the Recovery Narrative are so self-evident that they are seldom if ever made explicit within the recovery literatures. However, as we aim to show, critically untangling these interlocking convictions is essential to developing a more nuanced account of the production, performance and consumption of this genre.

## The Recovery Narrative: Four Unspoken Tenets

### “The Recovery Narrative Speaks to All Human Experience”

We have been careful to isolate some of the defining formal and rhetorical features of Recovery Narrative, delimiting this particular form of storytelling from the myriad forms of self-expression available to us at this socio-historical juncture. Our analysis of the founding assumptions of this genre starts by locating it within a much broader set of culturally specific logics. These are what philosopher Galen Strawson calls the psychological and ethical narrativity theses. According to Strawson:

The psychological Narrativity thesis is a straightforwardly empirical, descriptive thesis about the way ordinary human beings actually experience their lives. This is how we are, it says, this is our nature.

The psychological Narrativity thesis is often coupled with a normative thesis...the *ethical Narrativity thesis*. This states that experiencing or conceiving one’s life as a narrative is a good thing; a richly Narrative outlook is essential to a well-lived life, to true or full personhood. (Strawson 2004, 428)

Controversially arguing against a voluminous literature, extending from philosophy and psychology across the humanities and social sciences, Strawson dismisses as “mistaken and potentially pernicious” the “ideal of control and self-awareness” that underpins our collective enchantment with narrative models of the self:

The aspiration to explicit Narrative self-articulation is natural for some – for some, perhaps, it may even be helpful – but in others it is highly unnatural and ruinous. My guess is that it almost always does more harm than good – that the Narrative tendency to look for story or narrative coherence in one’s life is, in general, a gross hindrance to self-understanding: to a just, general, practically real sense, implicit or explicit, of one’s nature.(Strawson 2004, 447)

While Strawson’s claims concern any story we might tell about our lives, they arguably take on a greater moral and political significance in contexts where “explicit Narrative self-articulation” is a social, institutional or therapeutic imperative, and for people whose experiences place them at particular risk of not withstanding its potentially “highly unnatural and ruinous” effects. As Woods (2011, 2012a) has shown, Strawson’s critique of narrativity and his analysis of “episodic” and non-narrative modes of being have important implications in the context of illness for precisely these reasons. In the case of the Recovery Narrative, the genre’s claim to be a mode of authentic self-expression goes beyond being a sincere and factually accurate (if necessarily selective) description of an individual’s experience of recovery. In a deeper sense it asserts a particular discursive form as expressive of our “true nature” as narrative selves.

If the drive towards narrative self-expression is *not* universally shared, if there are “deeply non-Narrative people” and “good ways to live that are deeply non-Narrative” (Strawson 2004:429), what are the consequences of valorising a very narrow and circumscribed narrative form as something to which all people experiencing mental distress should aspire? Is there a danger, highlighted by Brian Schiff, that “we are reifying a Western, arguably middle and upper class, concept as a universal mode of shaping and articulating subjective experience”(Schiff 2006:21)? It is hard to overlook the fact that Recovery Narratives in current Anglophone circulation are not, generally, as representative of the population as is implied by their advocates.^8^ The Recovery Narrative can serve to whitewash madness and the ways “it is graphed on bodies differently,” and risks “erasing how systems of power require one another, and the material consequences of such biopower” (Voronka 2016b). The Recovery Narrative promoted by national mental health campaigns frequently prioritises, for example, younger voices and photogenic faces. We tend to hear most from white, often middle-class cis women and men at conferences. One reaction to this might be to increase “diversity” and “representation” amongst speakers. However, this does not take into account wider issues around the homogenisation of “lived experience” and madness (Voronka 2016a; Jones and Kelly 2015). Whilst emphasising heterogeneities risk “strengthening and legitimizing a hierarchy of suffering or marginalization within madness”, with onerous consequences for organising and coalition-building, not doing so allows “questionable practices of over-reach… to continue unchecked” (Jones and Kelly 2015:54).

The Recovery Narrative is seen as beneficial because it “gives voice” to those who have been systematically disempowered. The struggle to have the voices and stories of psychiatric survivors heard and framed as politicised accounts and loci of knowledge, instead of meaningless ramblings, is central to the psychiatric survivor movement (Costa et al. 2012). It should follow that the Recovery Narrative, which positions the individual as the agent of her own story, rather than as the recipient of clinical care, consumer of mental health services, or object of research, is by definition empowering. However, while it invests certain experiences with meaning and value, the Recovery Narrative can, like other narratives, also silence and exclude, by privileging and valuing certain kinds of reasoning and knowledge (Fitzpatrick 2016a:266).

This is thrown starkly into relief when we consider stories which do not fit comfortably within this genre: stories which fail to achieve recognition as a Recovery Narrative because they break formal conventions, or risk and even embrace ambivalence, ambiguity or abjection; stories which might offer insight into the “wrong” set of circumstances, issue the “wrong” kind of call to action or aim for the “wrong” set of goals (Rose 2014:217). Stories of psychiatric neglect and the struggle to access psychiatric care (Spandler 2017; Kelly 2016); experiences understood within a framework of neurodiversity (Jones and Kelly 2015); and cyclical experiences of relapse and readmission (Walker 2014) are less likely to be acknowledged as Recovery Narratives, not simply because they foreground different experiences but because they instantiate different relations between narrators and their interlocutors. What is worrying is that it is not just the story which can therefore be excluded from contexts in which the Recovery Narrative prevails, but also the would-be story-teller. The complex intersections of, amongst others, social class, disability, access, precarity and racialisation trouble the Recovery Narrative: the poor, socially marginalised or those who continue to need support and services (Kelly 2016), may, as individuals who challenge more homogenised survivor identities, find themselves cast adrift (Jones and Kelly 2015). The Recovery Narrative can thus occlude those stories and silence those voices which do not fit its strict parameters; insofar as it becomes the dominant mode of representing experiences of mental distress, it silences those who identify as “unrecovered” (Recovery in the Bin n.d.), reject the Recovery Model, or identify as disabled; those long-term service users who still need services and support; and those who have taken their lives.

### “The Benefits of Recovery Narrative for the Storyteller Greatly Outweigh Any Harms”

A second unspoken tenet of the Recovery Narrative, and a particularly striking instantiation of the ethical narrativity thesis, is that the representation of experience through this genre is intrinsically good for the storyteller. The healing and transformative powers of “telling your story” headline the how-to guides while the potential costs and negative consequences of disclosure are minimised or downplayed [in the SAMSHA guide, for example, only two pages in seventy refer, loosely, to potential drawbacks (2017:15, 18)]. By contrast, activists in the survivor movement have called attention to the multiple (if sometimes unintended) harms which can arise not simply from disclosing one’s experiences of mental distress, but of doing so through the form of Recovery Narratives shared in mental health settings. Documenting a community event held in Toronto in 2011 which was “organized in response to the appropriation and overreliance on the psychiatric patient ‘personal story’” Lucy Costa and colleagues offer a powerful analysis of the coercive logics and potentially damaging effects of sharing a Recovery Narrative (Costa et al. 2012:85), and six cautionary tips for those brave enough to do so:

- Participation is voluntary. You can always say no.
- Ask yourself, who profits from you telling your story?
- What purpose does personal story sharing serve?
- How do large organizations use stories to make material change?
- Story telling as an exercise of labour/work. Do you get paid?
- The internet lasts forever. Because of the technology available today, your interview or story will likely be accessible to the public for a very long time. That includes future employers and landlords. (Costa et al. 2012:94)

In particular, the emotional labour involved in producing and (re-) performing a Recovery Narrative profoundly problematises an uncritical celebration of their therapeutic and even political benefits. Critical disability and mad studies scholar Jijian Voronka focuses on the emotional labour involved in performing what she calls the “authenticity paradox”:

Either we are “too professional,” and thus cannot effectively represent abjection, or we are “too abject” and thus incomprehensible to respectability. This is a performance that as “people with lived experience” we must balance. As effective representatives, we must learn how to manage and present as both, as needed: When to bring our abject out, when to perform White civility. It is also an untenable position to hold (Voronka 2016a, 213).

The Recovery Narrative cannot, in its tone, content or delivery, be too disturbing, too dark, too angry; nor can it be too light, frivolous, or happy. It has to offer enough shade for the light of hope to be foregrounded, but not too much as to shroud it. It is for the narrator to manipulate her experiences but also her identity in ways which meet these conventions. She needs to judge what can be shared and what cannot, and calibrate her emotions and the rendering of her emotions so that she is angry enough, but without becoming the “angry consumer” (Jones and Cutler 2018). The Recovery Narrative confers authenticity and authority on the narrator as “peer”, as having “expertise by experience”(Noorani 2013); stepping outside of its generic boundaries risks confounding these identities (Voronka In Press).

A further danger is of the Recovery Narrative is that it becomes and is received as finished, definitive, “on the record”—rather than being dialogic (Frank 2010); open to flow, change and revision. The potential disconnect between the person’s story and her circumstances can trouble claims to authenticity embedded in the Recovery Narrative, creating a gap which can widen with each retelling:

In repeatedly telling my story, there has been an inevitable loss of ownership. There is little space for my narrative to develop, as personal narratives must: in a public arena, it is hard to give expression to doubt, contradiction, and ambiguity. I must adopt a language that is clear, direct and easily comprehensible: this is not always my preferred language. There are parts of my story that I can no longer distinguish from the telling. (Shaw 2016, 278)

Turning experience into a coherent story is inherent to the technologies of recovery, but at what cost? There is a teleological quality to the Recovery Narrative which propels us towards a transformed and renewed self (Frank 2013). However, this can be enacted at the expense of the ineffable, the inexpressible, those experiences for which we do not have the words, the formless and the meaningless (Woods 2012a).

Indeed, perhaps the imperative to see oneself as possessing a Recovery Narrative might actually prevent self-understanding, authenticity and meaning-making. This might seem especially concerning given these qualities are ostensibly the purpose of recovery: here the Recovery Narrative as a fixed account of a self-in-process is in tension with the prevailing idea of recovery as an ongoing “journey.” Paradoxically, the Recovery Narrative can effectively rob the speaker of agency even where it demands particular forms of agency (heroic self-determination) be asserted at the level of thematic content. Again, the constraints of the form strongly influence what is heard by the interlocutor:

[H]owever hard I try to frame my peer narrative as something other than a personal family tragedy of weakness, poverty, and mental illness, metanarratives of heroic overcoming through resilience and recovery strategies prevail. The conditions under which I am heard outweigh and overwhelm me. (Voronka In Press)

Voronka’s narrative actively seeks to disrupt the Recovery Narrative, and yet she is still subject to her interlocutors, exposed, raw and vulnerable to their framing: “I am the stranger revealed”(Voronka In Press). If the Recovery Narrative has more agency than the individual recovery-story teller, it is not hard to see how its conventions could feel constrictive, coercive or even like a “vehicle of oppression” (Gabriel 2008:169).

### “The Recovery Narrative Always has Wider Social Benefits Beyond the Individual Story Teller”

While the Recovery Narrative is, in the main, a recounting of the past, its main preoccupation is in fact futurity, specifically the imagining of various futures in the spheres of mental health. The Recovery Narrative is goal-oriented and seeks not just to inspire the narrator and the interlocutor, but also to transform the wider mental health landscape of policy, services and communities. It works in part because of its resonance—it arouses emotions and imagination concerning how things could be different. However, its preoccupation with wider goals and futurity has caught the attention of critics who argue that this positions it explicitly within a neoliberal framework.

It has been argued extensively that recovery policy privileges an ideal of recovery related to certain ideals of neoliberal citizenship,^9^ and in so doing “reduces the horizon of possibilities for enacting recovery” in diverse ways (Fisher and Lees 2016:601). The most persuasive and oft-cited of these critiques maintain that there has been an unholy alliance between the modern recovery movement and the neoliberal restructuring of society (Teghtsoonian 2009; Howell and Voronka 2012; Morrow 2013; Esposito and Perez 2014; Harper and Speed 2014). McWade (2015), for example, argues that recovery-as-policy in the UK is a form of neoliberal state-making which window-dresses the restructuring of the relations of domination implicit in mental health services; policing the crisis of faith in psychiatry brought about (in part) by critiques of psychiatry and user/survivor and allies’ activism. As a key “technology of recovery,” the Recovery Narrative focuses attention on individuals’ “recovery journeys” rather than the “social, political, cultural and economic context in which people become mentally distressed and recover” (Morrow 2013:325):

[i]ssues of systemic poverty and discrimination, an appalling lack of choice in services, and mistreatment are conveniently left out of the story. Favoured stories feature the uplifting message that with a little hard work and perseverance, you too can be cured. Common themes include: How this or that service saved my life; how this or that medication saved my life; and how this or that pursuit of a normal existence saved my life. (Costa et al. 2012, 89)

Here as in many clinical settings, the Recovery Narrative’s measure of success is the extent to which it can offer “hope” to other service-users and survivors, carers and mental health professionals. However, the concept of “hope”—like “recovery” and “resilience”—is so often regarded as self-evidently desirable that there is little critical discussion of its potentially negative impacts or the ways in which it is used to further a neo-liberal agenda (Ehrenreich 2010; Berlant 2011; Friedli and Stearn 2015). The Recovery Narrative becomes the principle vehicle through which a particular kind of hope is linked to entrepreneurial (Scharff 2016) future-oriented, outcome- and goal-focused modes of subjectivity which are tied to the “imperatives of economic participation,” productivity and “the ability to flourish financially” (Fisher and Lees 2016:603, 604). While hope appears self-evidently a “good thing,” false expectations may lead to a form of what Berlant (2011) has called “cruel optimism.”

It is not controversial to argue that by abstracting the individual from their immediate social network and wider social context, and in turn abstracting mental health from wider social, cultural and affective determinants of health, the Recovery Narrative might foreclose the collective changes for which many argue. Less attention has been paid to stories recognised as Recovery Narratives within the survivor movement—whether of overcoming trauma and escaping psychiatry, rejecting treatment, or finding spiritual renewal—which might also preclude certain complexities. Much like mainstream Recovery Narratives, they can exemplify the psychological narrativity thesis and lay claim to being “transcultural, transhistorical truths of the human experience” (Woods, 2011), presenting the individual as bounded, responsible and autonomous and framing adversity as an impediment to be overcome. The unintentional effect can be to depoliticise madness and to minimise the structural barriers which might obstruct it, thus ironically preserving the status quo.

If the common themes of individual agency and futurity render both mainstream and survivor Recovery Narratives vulnerable to claims of neo-liberalism, the “structured situation of address between author and reader” (Woods 2012b:43) fundamentally unifies them as a genre of insight and of inspiration. The Recovery Narrative instantiates the position of the collective or organisation which commissioned them, whether that is recovery-as-policy, medical compliance, spiritual emergence, escape from services or a rejection of psychiatry. As well as telling an individual story, they voice a wider political framing of mental health. It is not that the narrator is duped or disempowered into “selling recovery”, rather that she has a personal stake in the ideas she espouses, as they have been central to her survival. Part of the function of the Recovery Narrative, therefore, is as a rhetorical device to unite interlocutors in their mission, to sustain them in their pursuit, to shore up their activities and political persuasion, and to prove that their shared position is correct. The political effects of Recovery Narratives are therefore only as benign as the context in which they are materialised will allow.

### “The Recovery Narrative Should not be the Focus of Critique”

These three major guiding assumptions may help explain why, despite a vast and vibrant critical literature on recovery, there has been a conspicuous lack of critical engagement with the Recovery Narrative as genre. One striking exception to this is Lucy Costa and colleagues’ account of Recovery Narrative as a type of “‘disability tourism’ or ‘patient porn’” (Costa et al. 2012).^10^ The provocative term “porn” is used to examine how stories are told and how they are heard (Voronka In Press), how they might be commodified, how they are performed, how they are consumed, and how they are valued. Costa et al. used the term in their “Hands off our Stories” event to refer to “a modern day voyeurism whereby, in listening to a cast of characters, spectators continue to justify the ‘otherness’ of madness while curbing the watcher’s anxiety” (Costa et al. 2012:86,92).

While there has been considerable scholarly engagement with notions of “disability porn” and “poverty porn”, the framing of Recovery Narrative as “recovery porn” or “patient porn” has largely been largely ignored in scholarly work. Costa et al. acknowledge that the term may be seen as highly offensive, graphic and provocative, but they highlight its capacity to provoke resistance and critique, naming “a phenomenon that other marginalized communities can relate and respond to” (Costa et al. 2012:95). What interests us here is the taboo that surrounds these claims. To be clear, we are not arguing the Recovery Narrative is “recovery porn” and we have never experienced any testimony as such. Rather, our discussion of it calls attention instead to the very real political and personal sensitivities which surround Recovery Narrative as a genre, and a widespread reticence to engage with these. If we cannot discuss and analyse the Recovery Narratives as a genre (as that is what is at stake), then how do we raise concerns about the forces behind it? And yet- how do we disentangle Recovery Narrative as a genre from the individuals and communities who may benefit from the telling of individual recovery stories?

## Conclusion: A Call for Alternative Frameworks

In the way that it has been shaped by mainstream mental health services and by many in the psychiatric survivor movement, recovery is inextricably bound up with individuals’ stories. Our argument in this paper is that recovery—as policy and as political rallying point—is in fact currently materialised and enabled through only a very particular type of story: the Recovery Narrative. We have suggested that the concept of genre is helpful, both in identifying some of the formal features of Recovery Narrative and in locating these within specific contexts and sets of social relations. One of the contributions of this paper, then, is to identify the Recovery Narrative *as* a genre, that is, to show that only a narrow sub-set of the stories it is possible to tell about recovery (can) function as Recovery Narrative. This is important because as a *dominant* genre within mainstream mental health services *and* many parts of the survivor movement, the Recovery Narrative constrains and restricts which experiences can be shared.

The concept of genre is not intended to be normative: we are not arguing that the generic conventions of Recovery Narrative are good or bad; or that making use of these conventions, consciously or implicitly, is good or bad, institutionally or individually. We do believe, however, that the centrality of the Recovery Narrative to recovery-as-policy *and* to the vision of recovery promoted by many in the service-user/survivor movements means that, as a genre, it should not escape critical scrutiny. In this sense, understanding the Recovery Narrative as genre is not an end in itself so much as a step towards opening up broader dialogue, highlighting existing alternatives and imagining otherwise (Fisher and Lees 2016).

We want to conclude by exploring just some of the alternative contexts in which experiences of madness and mental distress, survival and flourishing, are shared. If the Recovery Narrative is enacted within specific sites and spaces, ranging from the classroom of the Recovery College to the high-profile videos of anti-stigma campaigns, it is worth considering what contexts might support alternative genres are enabled and accepted. These are more likely to exist in counter cultural spaces at a critical distance from mainstream mental health services that do not have to answer to recovery “outcomes.” Whereas organisations such as Alcoholics Anonymous require members’ testimony to be presented in relation to fixed stages of recovery, grassroots self-help or peer support groups can, for example, encourage a more fluid and iterative sharing of individual experiences (Noorani 2013; Dillon and Hornstein 2013; McCormack 2015). At their best, as “epistemic communities of problem solvers” which honour “deep experiential knowledge” (Noorani, Karlsson, and Borkman 2019), they actively embrace and honour silence, ambiguity, contradictions and uncertainty. In opposition to the fixity of more conventional and constrained illness narratives and patient identities, there is just being, and being with; people holding space between the urge to story one’s experiences, and the difficulties of so doing. By privileging un-knowing and uncertainty, such groups are able to perform an epistemological function—critically interrogating both scientific knowledge (Emerick 1996; Faulkner and Basset 2012) and narrativity itself- while at the same time fostering different kinds of social relations which do not depend on insight or inspiration as we have defined them here.

Just as different spaces—physical and discursive—afford different opportunities for storytelling, so too do different narrative formats. Again, there are powerful examples from the survivor and mad studies movements of genre-defying narratives of mental distress and recovery. To list but a few: multi-voiced, politically charged and frequently satirical publications such as *Mad Pride* (Dellar, Curtis, and Leslie 2003)*, Asylum* and other “zines”; blogs such as *purplepersuasion*, *Behind the Label* and *My C*-*PTSD Recovery Journal* which foreground complexity, change and dialogicity (Walker 2013; Waddingham 2012; Wilson 2017); documentary films like *In the Real* (McCormack 2015) which explore shared meaning-making across time; irreverent podcasts like *Coffee and Psychosis* (“Coffee and Psychosis – a Podcast” 2017); exhibitions emphasising a diversity of perspectives such as *Hearing Voices: Suffering, Inspiration and the Everyday* and *Mr A Moves in Mysterious Ways* (Hearing the Voice 2016; Tilley and Johnstone 2017); and even online games such as Depression Quest which open up very different structures of identification with respect to mental ill health (Quinn 2013). These narratives, many of which do not simply embrace or embody but also theorise their own open-endedness, reinforce calls by medical humanities scholars to consider the potential of short-form and avant-garde forms (Magi, Jones, and Kelly 2016; Salisbury 2016; Wasson 2018) in illuminating aspects of experiences which are painful and difficult to articulate.

Finally, as well as attending to the multiple spaces and modalities through which individuals’ accounts of madness, distress and mental illness are shared, we might venture a step farther in exploring ways of conceptualising and enacting passages through suffering which do not begin and end with the individual. One striking feature of the Recovery Narrative—so consistent and apparently self-evident that we have not remarked on it until now—is that is bound to and by the first-person singular; its efficacy is indexed to the experience of a single individual. What might be opened up, revealed or foreclosed in telling a recovery story in the first-person plural? Could narratological analysis of “we-narration” (Richardson 2006; Bekhta 2017), particularly with respect to testimonies of shared traumatic experience (Dwivedi and Nielsen 2013), offer promising avenues for exploring the storytelling made possible by collective voices? This might this, in turn, connect with recent work in geography and anthropology on “relational” models of recovery (Price-Robertson, Obradovic, and Morgan 2017; Price-Robertson, Manderson, and Duff 2017) attuned to notions of affective atmosphere (Duff 2016).

We hope this analysis, and the wider interdisciplinary and survivor-produced scholarship upon which it draws, can increase awareness of the ways in which one particular genre of storytelling, the Recovery Narrative, works and with what effects. There is good reason to continue to tell, listen to and celebrate Recovery Narratives, in a range of contexts. And there will also, we think, be good reason to question whether as the *dominant* narrative form in those contexts it may be considerably limiting what it is possible to see, hear, acknowledge or act upon.

## Footnotes

1 As we are careful to note here, and will go on to explain in more detail, the genre we are describing is not limited to published memoirs or other written accounts. More typically, the Recovery Narrative is performed or put to use within a specific context such as the opening of a conference, launch of a new policy, training of mental health professionals or delivery of peer support. There are many Recovery Narratives in circulation in public as well as clinical settings (see the Narrative Story Bank (Scottish Recovery Network n.d.) and Nottingham University’s NEON Collection (Narrative Experiences Online n.d.) for examples) but perhaps Eleanor Longden’s TED talk ‘The Voices in My Head’ (Longden 2013), viewed over 4.5 million times, is the best exemplar of the genre as we will go on to define it.

2 Indeed, so well-rehearsed is this formulation that in her deliciously terse editorial “The Mainstreaming of Recovery,” Professor of User-Led Research Diana Rose refused to quote Anthony directly (Rose 2014).

3 On this last point, the authors remain somewhat sceptical of participants’ insistence that the “Scottish” model of recovery differs from its US and New Zealand counterparts, noting that: “One function of the collection of Scottish recovery narratives thus appears to have been to help to strengthen an indigenous recovery movement by articulating a shared Scottish identity around recovery in contrast to a fictional American ‘other’” (2011:p3).

4 One could argue on this basis that for activist collectives such as Recovery in The Bin who espouse the social model of disability and advocate for more statutory provision, even the original and radical consumer-led definition of recovery might be problematic.

5 Writing in 2007, Lakeman, Walsh and McGowan suggested that a hierarchy has emerged in which some service-users have attained “professional”, “corporate” or “celebrity” status through sharing their narratives of mental distress. For individuals so recognised, “the more noxious the experience, for example being incarcerated, or more dramatic the story, for example of psychosis or suicide attempts, the greater the perceived legitimacy. Once these credentials are established they do not need to be renewed and, indeed, people often provide an account of recovery, transcending the need for mental health services or overcoming adversity. They typically project remarkable charisma, confidence, and talents [… and the] authority of the celebrity often extends well beyond their knowledge, expertise or experience.” (Lakeman, Walsh, and McGowan 2007, 15–16). We have quoted their work at some length in order clearly to distinguish our own analytic enterprise, focussed on the structure and type of narration, from one which targets individual narrators themselves.

6 Indeed, it would be almost unthinkable to bring together a collection of narratives in this genre without an editorial steer; whatever the desire for polyphony, a certain formal homogeneity must prevail. As the editors note: “Our initial request was for people to write their own stories and this resulted in some very fine narratives, but not enough to meet our target of fifty. We therefore took the step of offering to work with people and we interviewed many of the people whose stories are to be read in this book. We edited the interviews down to a maximum of four A4 typed pages, focusing on what was said about recovery, but keeping the words used by the voice hearer in the interview.” (Romme et al. 2009:5)

7 Significantly, recognising a story as a Recovery Narrative might come as a consequence of failing or choosing not to hear the messy, awkward or upsetting elements of an individual’s story, and therefore not congruent with the intentions of the speaker.

8 Perhaps because Recovery Narratives come into circulation through a variety of contexts—including Recovery Colleges, mental health charities, NHS and independent training sessions and survivor advocacy groups—there has been little if any systematic analysis of whose stories they tell. However, two examples discussed at length in this article are illustrative of wider trends: of the 67 contributors of Recovery Narratives to the Scottish Recovery Network project, only 3% were from a “non-white ethnic origin” (Brown and Kandirikirira 2007:18), and while ethnic origin is not formally reported by the 50 contributors to *Living with Voices*, overwhelmingly they appear to be white British or Dutch(Romme et al. 2009:102–103). The under-representation of black and minority ethnic communities in recovery literatures and practices of all kinds is an ongoing concern (Kalathil 2011).

9 “Neoliberalism,” as Naomi Schiller argues, is “not simply a revival of classical liberal political economy that advocates for a small state and a free market, but represents instead the imposition of values of privatization, market freedom, and atomized individual responsibility to *all* spheres of human life” (Schiller 2015:12).

10 In the notes to the paper, Costa explains that she first heard the term from a fellow activist, Lana Frado, in 2005, who references Heather McKee as coining the term (Costa et al. 2012:99). The first reference to it in the UK is from Pembroke (Pembroke 2004).
